# How often do nurses suspect violence and domestic violence in local emergency medical communication centre? A cross-sectional study

**DOI:** 10.1080/02813432.2022.2097615

**Published:** 2022-07-11

**Authors:** K. Steen, K. Alsaker, G. Raknes

**Affiliations:** a National Centre for Emergency Primary Health Care, NORCE (Norwegian Research Center), Bergen, Norway; b Minor Injury Department, Orthopedic Division, Haukeland University Hospital, Bergen, Norway; c Department of Welfare and Participation, Western Norway University of Applied Sciences, Campus Bergen, Bergen, Norway; d Raknes Research, Ulset, Norway

**Keywords:** Violence, screening, Norway, emergency primary care, out-of-hours, nurse, triage

## Abstract

**Objective:**

To assess the extent of violence that is revealed by screening at first contact with a local out-of-hours emergency medical communication centre (LEMC; Norwegian ‘Legevaktsentral’).

**Design:**

Cross-sectional study.

**Setting:**

Arendal LEMC, covering 10 municipalities in south-eastern Norway. All contacting patients (telephone or personal attendance) were asked by nurse whether the encounter was related to violence.

**Subjects:**

All first patient encounters at Arendal LEMC.

**Main outcome measures:**

Number and proportion of cases where the nurses suspected violence, both domestic violence and other violence. Incidence rate of violence, age and gender distribution of patients, time of day and reason for encounter.

**Results:**

Violence was suspected in 336 of 103,467 first patient encounters (0.3%), of which 132 (0.1%) was domestic violence. Patients were female in 50.6% of all violence cases, and in 79% of domestic violence cases. Incidence rates were 137 per 100,000 person-years for all violence, and 53 for domestic violence.

**Conclusions:**

This study indicates violence may be revealed in three of 1000 first encounters to an LEMC when nurses screen systematically for domestic or other violence.

**Key points**
    Violence as underlying reason for encounter with primary care emergency health services is probably often not discovered by health personnel.
  • We examined how often nurses reveal violence upon first contact when systematically asking all patients.
  • Violence was suspected in 0.3% of cases, and domestic violence in 0.1%.
  • Among patients with disclosed domestic violence, 79% were female.

## Introduction

Violence is a serious health problem worldwide [1] that probably should receive more attention from healthcare professionals. Based on self-reported data on living conditions from Statistics Norway, 4.9% of women and 4.4% of men in Norway were exposed to violence or threats of violence during the preceding 12 months in 2018. These percentages have been stable over the last decades [2]. The majority of domestic violence victims are female, while street violence is most common among men [1,2].

Exposure to violence has profound and long negative health effects on the victims [3–5], and it seems plausible that there are great potential health benefits from detecting violence and implementing measures at an early stage. However, screening of violence, and especially domestic violence is controversial. This is mainly due to lack of evidence that screening leads to better outcomes for the identified victims. Usually, such screening is done during face-to-face meetings with health care professionals or by self-completed questionnaires at health care settings [6].

Only a minority of female victims of domestic violence are recognised by health care professionals during their visits to at emergency rooms in the United States [7]. Systematic screening of violence at the patients’ first encounter with the triage nurse at the emergency primary care has rarely been studied. This could be an effective way to disclose cases that would otherwise have remained undetected in a following consultation with a physician. We wanted to quantify the potential of minimal intervention screening by triage nurses in out-of-hours (OOH) emergency primary care. The aim of this study was to determine how often violence is suspected by triage nurses upon first patient contact with emergency primary care in Norway, and how this was associated with patient and contact characteristics, reason for encounter and type of violence.

## Methods

### Study design, setting and participants

In Norway, primary care has an important role as gatekeeper for admissions to hospitals even in emergencies. All municipalities have OOH emergency clinics (‘legevakt’) staffed by general practitioners (GPs). Most often patients must make an initial contact with triage nurses in the local emergency medical communication centre (LEMC, ‘legevaktsentral’). The triage nurses will then decide whether an appointment with a doctor is necessary, or if not urgent, the inquiry can be closed by giving the patient advice without face-to-face consultations. These first encounters are usually phone calls, but at some LEMCs it is possible to show up in person.

We performed a cross-sectional study, based on all patient contact with Arendal LEMC in Arendal, Norway. The LEMC handles all inquiries by telephone or personal attendance to the OOH emergency primary care clinic serving Arendal and nine surrounding municipalities covering 5634 square kilometres, with populations ranging from 1323 to 44,313, 94,537 (2016) in total. We collected data from 1 January 2014 to 31 December 2017.

All patients that contacted the LEMC in the study period were eligible for the study.

### Variables

Main outcomes were proportion of suspected violence (total, domestic and other violence) among inquiries to the LEMC, and the incidence of such cases in the population in males and females. Secondary outcomes include proportions and odds that violence was suspected among patients within different age and gender groups. Frequencies of injuries and the different ICPC-2 reasons for encounter (RFE) chapters among violence suspected inquiries was another secondary outcome.

Secondary outcomes also include incidence rates of violence, proportions and odds ratio (OR) that different types of violence were suspected among patients within different age and gender groups. Time of encounter and RFE using ICPC-2 codes and triage nurse response were also secondary outcomes.

### Data sources

Data were collected from a database, the so-called ‘Watchtower project’, which is a sentinel network of representative OOH emergency primary health care activity in Norway. The development and implementation of this project has been described in detail elsewhere [8]. An online data collection tool was developed using the cloud-based, low-code app development platform Zoho Creator. For every first contact by telephone or personal attendance, trained staff (nurse or other) was instructed to record relevant information, including age, gender, mode of contact, urgency level according to the Norwegian Index for Medical Emergency Assistance [9], first action taken, and ICPC-2 (International Classification of Primary Care) code according to the official reason for encounter manual [10].

The Norwegian Index for Medical Emergency Assistance is a widely used emergency triage tool used in Norway. According to this tool, the patients are triaged as acute (or red response, potentially life threatening), urgent (or yellow response, acute, but not life threatening) or not urgent (or green response, can wait).

From 2014 on, violence has been among the information to be collected in Arendal LEMC. Upon first encounter with any patient, the LEMC nurse was instructed to routinely ask all patients about if the inquiry had any relation to violence in general regardless of reason for encounter, and if so, whether it was domestic violence. There were no standardised question formulations, but the nurses had to fill in fixed alternatives in the registration tool. The alternatives available were: ‘Ikke mistanke om vold’ (‘No violence suspected’), ‘Mistanke om vold i nær relasjon’ (‘Domestic violence suspected’), and ‘Mistanke om annen vold’ (‘Other violence suspected’). ‘No violence suspected’ was default option. We use the term ‘domestic violence’ throughout this paper. The term used by the nurses (‘vold i nær relasjon’) directly translated means ‘violence in close relationship’. Often this means intimate partner violence, but it may also include family members and close friends both within and outside one’s own household.

The staff received training in how to disclose domestic violence before the project began. The Watchtower registration was done only once for each patient inquiry and this was done at first patient contact with triage nurse at the LEMC, regardless of type of contact (including telephone and personal attendance).

Information was only collected by triage nurses at first contact. No information about further examination or treatment by physicians or other health personnel that with greater certainty could confirm that the patient was actually exposed to violence was recorded.

### Statistical methods

Incidence rates were based on gender specific population data from Statistics Norway for the 10 municipalities (population: 94,537 (2016)) served by the ED. The incidence presented is annualised incidence 2014–2017, and this was used to calculate incidence rate ratios (IRRs) with 95% confidence intervals to compare males and females [11]. Odds ratios with 95% confidence intervals were calculated to quantify odds that violence was suspected in females compared to males, and for different age and gender groups compared to males aged 30–66. The age grouping was based on the minimum age for criminal responsibility in Norway (15 years) and the retirement age (67 years). Young adults (16–29 years) and older adults (30–66 years) were also defined as age groups.

In principle, all inquiries were to be recorded, but unavoidably, some cases have not been captured. Reasons include that the nurses in periods with high workload were not able to perform the Watchtower registration, and sometimes it was forgotten. The incidence rates were adjusted for underreporting according to a model used in another study based on Watchtower data [12] and in reports presenting data for the project [13]. The number of cases for each year was multiplied by an adjustment factor based on the ratio between reimbursement claims to HELFO (The Norwegian Health Economics Administration) and Watchtower registrations and historic deviations in this ratio. For technical reasons, data from December 2016 were lost, and for 2016 the annual incidence adjusted for these lacking data. We assumed that the proportion of data for December 2016 was similar to the average proportion for December of the other years analysed.

Excel was used for the statistical analyses. The results are given as absolute numbers, percentages with 95% confidence intervals for proportion or incidence rates.

### Ethics

The Watchtower project has been approved by the Norwegian Social Science Data Services, and by the Privacy Ombudsman for Research for Uni Research (from 10/1/18 NORCE Research). No patient identifiable data were recorded at any time, and approval from Regional Committee for Medical and Health Research Ethics was neither necessary nor relevant. The approvals do not allow sharing of underlying data.

## Results

### Descriptive data

We included 104,467 inquiries in our analysis. Baseline data are given in Table 1. Age and proportion of females were lower, and the proportion of yellow urgency level was higher in cases with violence compared with no violence. Gender was unknown in 33 cases, and age was unknown in 438 cases. Estimated underreporting varied from 25.0% in 2017 to 40.2% in 2016.

**Table 1.** Baseline data.

	Violence		No violence	
*N* (%)	336	(0.3)	104,091	(99.7)
Females, *N* (%)	170	(50.6)	56,253	(54.0)
Age				
Average (SD)	38.2	(76.1)	36.5	(26.6)
Median (IQR)	29	(20–45)	33	(14–57)
Urgency level				
Yellow, *N* (%)	192	(57.1)	29,131	(28.0)
Red, *N* (%)	9	(2.7)	3812	(3.7)

SD: standard deviation; IQR: interquartile range.

*Urgency levels*. Yellow: acute, but not life threatening; red: potentially life threatening.

### Main results

Table 2 presents the number of and incidence of cases where violence was suspected by gender and type of violence, and in Table 3 in different age groups. Violence was suspected in 0.3% (*n* = 336) of all inquiries, and domestic violence in 0.1% of all inquiries (*n* = 132). For all suspected violence combined, we found a nearly equal number of suspected male (49.4%) and female victims (50.6%). Domestic violence was predominant among suspected female victims of violence (61.2%), while males dominated among suspected victims of other, non-domestic violence (83.1%). For both males and females, the odds if domestic violence suspected were significantly higher among both male and female 15–29 years of age compared to males aged 30–66. The odds of suspected domestic violence were approximately fourfold higher among all females aged 15–66 compared to males 30–66. For other violence, OR was lower for all females than males age 30–66. Young adult males were overrepresented when it comes to other violence. Both the youngest and the oldest patients had a lower occurrence of suspected violence.

**Table 2.** Inquiries to Arendal out-of-hours local emergency medical communication centre (LEMC) where the nurse suspected violence, by gender and type of violence.

		All violence		Domestic violence		Other violence		No violence suspected	
*N* (%)	All	336	(0.32)	132	(0.13)	203	(0.19)	103,884	(99.68)
	Male	166	(0.35)	28	(0.06)	138	(0.29)	47,827	(99.65)
	Female	170	(0.30)	104	(0.19)	66	(0.12)	56,024	(99.70)
	Gender unknown	0	(0.00)	0	(0.00)	0	(0.00)	33	(100.00)
OR (95% CI)	Male = ref.	0.87 (0.71–1.08)		2.18 (2.09–4.82)		0.41 (0.30–0.55)		1.00 (0.98–1.02)	
Incidence	All	136.8		52.9		83.9		41866.0	
	Male	136.5		22.6		114.0		38366.7	
	Female	137.0		83.5		53.6		45351.9	
IRR (95% CI)	Male = ref.	1.00 (0.91–1.11)		3.70 (3.47–3.94)		0.47 (0.43–0.51)		1.18 (0.20–7.10)	

Odds ratio (OR) with 95% confidence intervals (95% CI) that violence is suspected in females compared to males (reference). Incidence per 100,000 person-years. Incidence rate ratios (IRR) with 95% confidence intervals (95% CI), male is reference.

**Table 3.** Inquiries to the LEMC where the triage nurse suspected violence by age, gender and type of violence.

		Male			Female			Both genders		
	Age	*N*	%	OR (95% CI)	*N*	%	OR (95% CI)	*N*	%	OR (95% CI)
All violence	0–14	14	0.10	0.25 (0.23–0.27)	14	0.11	0.27 (0.25–0.29)	28	0.11	0.26 (0.25–0.27)
	15–29	74	0.83	2.00 (1.95–2.06)	68	0.54	1.30 (1.27–1.34)	142	0.66	1.61 (1.59–1.64)
	30–66	74	0.41	REF	85	0.40	0.98 (0.95–1.00)	159	0.41	REF
	67+	3	0.04	0.10 (0.07–0.14)	2	0.02	0.05 (0.03–0.08)	5	0.03	0.07 (0.06–0.09)
	Unknown	1	0.51	1.22 (0.44–3.38)	1	0.44	1.07 (0.39–2.96)	2	0.46	1.12 (0.67–1.86)
Domestic violence	0–14	5	0.04	0.51 (0.39–0.67)	9	0.07	0.98 (0.81–1.18)	14	0.05	0.30 (0.27–0.32)
	15–29	8	0.09	1.23 (1.00–1.50)	38	0.30	4.15 (3.74–4.60)	46	0.21	1.18 (1.14–1.23)
	30–66	13	0.07	REF	57	0.27	3.74 (3.4–4.11)	70	0.18	REF
	67+	2	0.03	0.37 (0.21–0.65)	0	0.00	–	2	0.01	0.06 (0.04–0.11)
	Unknown	0	0.00	–	0	0.00	–	0	0.00	–
Other violence	0–14	9	0.07	0.19 (0.17–0.22)	5	0.04	0.12 (0.09–0.14)	14	0.05	0.23 (0.22–0.25)
	15–29	66	0.74	2.17 (2.10–2.24)	30	0.24	0.70 (0.66–0.73)	96	0.45	1.95 (1.91–1.99)
	30–66	61	0.34	REF	28	0.13	0.39 (0.37–0.41)	89	0.23	REF
	67+	1	0.01	0.04 (0.01–0.11)	2	0.02	0.06 (0.03–0.10)	3	0.02	0.07 (0.05–0.11)
	Unknown	1	0.51	1.48 (0.53–4.11)	1	0.44	1.30 (0.47–3.60)	2	0.46	2.00 (1.20–3.34)

Proportion of violence (%) of all cases within age and gender groups. Odds ratio (OR) with 95% confidence intervals (95% CI) that violence is suspected in each age and gender group compared with males age 30–66. For analyses on both genders combined, age group 30–66 is reference.

A total of 99 (29.5%) were personal patient attendances to the OOH emergency clinic and the rest was telephone inquiries. We found no significant difference regarding type of violence and mode of inquiry (personal attendance or by telephone).

### Incidence

Incidences of LEMC inquiries with suspected violence are presented in Table 2. The incidence of suspected violence was equivalent to one per 800 inhabitants, and one case per of domestic violence suspected in approximately every 1200 woman per year. The IRRs show that domestic violence was disclosed 3.7 times more often in the female population compared to males. On the other hand, other types of violence were discovered almost twice as often among men.

### Other outcomes

#### Age and gender

Median age was 30.5 years (SD 75.7 years) for females and 27.0 years (SD 76.8 years) for males. Suspected victims of domestic violence were older than suspected victims of other violence (domestic violence median age: females: 31.0 years and males 33.5 years versus other violence median age: females 28.8 years, males 25.9 years).

#### Time of encounter

The proportion of nighttime encounters was almost twice as large in cases with suspected violence than other cases (difference 18.9 percentage points, 95% CI 13.8–24.0) (Table 4). Most violence cases (65%) were not recorded at night. Daytime numbers are dominated by encounters during the weekend, because most patients use their GPs during normal daytime opening hours even in emergencies.

**Table 4.** Violence by time of day among 336 suspected victims of violence, percentages with 95% confidence intervals (CI).

	Day (8 am to 3.30 pm)			Afternoon/evening (3.30 pm to 11 pm)			Night (11 pm to 8 am)		
	*N*	%	95% CI	*N*	%	95% CI	*N*	%	95% CI
All violence	88	26.2	(21.5–30.9)	132	39.3	(34.1–44.5)	116	34.5	(29.4–39.6)
Other violence	57	27.9	(21.8–34.1)	69	33.8	(27.3–40.3)	78	38.2	(31.6–44.9)
Domestic violence	31	23.5	(16.3–30.7)	63	47.7	(39.2–56.2)	38	28.8	(21.1–36.5)
No violence suspected	38,650	37.1	(36.8–37.4)	49,205	47.3	(47.0–47.6)	16,275	15.6	(15.4–15.9)

### Reasons for encounter

Unspecific RFE dominated when the LEMC nurses suspected violence (Table 5). ICPC codes from chapter Z (social problems) and chapter A (general and unspecified) were most frequent. The most frequent single RFEs given were ‘Problems with violence/traumatic event’ followed by ‘Health problems/illness’.

**Table 5.** Reasons for encounter (RFE).

ICPC-2 chapter				Most frequent RFEs (ICPC-2)			
	*N*	%	95% CI		*N*	%	95% CI
Z: social problem	71	19.4	15.3–23.5	Z25: social problem/traumatic event	66	18.0	14.1–22.0
A: general and unspecified	60	16.4	12.6–20.2	A99: health problem/illness	44	12.0	8.7–15.4
L: musculoskeletal	55	15.0	11.4–18.7	S18: laceration	28	7.7	4.9–10.4
S: skin	44	12.0	8.7–15.4	N79: concussion	13	3.6	1.7–5.4
N: nervous system	27	7.4	4.7–10.1	L72–76: fractures	9	2.5	0.9–4.0
P: psychological	20	5.5	3.1–7.8				
Other chapters	24	6.6	4.0–9.1				
RFE missing	35	9.6	6.5–12.6				

Distribution of ICPC-2 codes among 336 suspected victims of violence, absolute numbers, percentages with 95% confidence intervals (CI).

### Triage

The suspected victims of violence were triaged as red/‘acute’ in nine cases (2.7%, 95% CI: 1.0–4.4%), yellow/‘urgent’ in 192 (57.1%, 95%: CI 51.9–62.4%) and green/‘non-urgent’ in 135 cases (40.2%, 95% CI: 34.9–45.4%). No significant gender difference regarding triage was found (data not shown).

The urgency levels were similar in both types of violence (domestic or other). The most frequent response after triage was referral to medical consultation at the primary care ED for both non-urgent (73.3, 95% CI 65.9–80.8%) and urgent cases (84.9, 95% CI 79.8–90.0%), while a majority of the acute responses resulted in an emergency dispatch of ambulance and medical doctor (five out of nine, 55.6%, 95% CI: 23.1–88.0%), the other four were referred to medical consultation at the ED (44.4, 95% CI: 12.0–76.1%).

## Discussion

### Statement of principal findings

In this study, where nurses in the LEMC screened for violence on first contact, we found that violence was suspected in 0.3% of all inquiries, and that nearly 40% of these were domestic violence. This is equivalent to an incidence of 53 per 100,000 person-years for domestic violence and 84 per 100,000 person-years for all other violence. Females were more than three times more likely to be victims of domestic violence than males, while the odds of other violence were more than two times higher in males. Violence was most often suspected among patients aged 15–29 and 30–66, and domestic violence was most frequent in women in the same age groups.

### Strengths and weaknesses of the study

The study was conducted by experienced nurses within the framework of a well-established project for collecting information about patients (‘the Watchtower Project’). In addition, the collection of data on violence was initiated by the employed nurses at Arendal LEMC, which may have contributed to commitment and less resistance to asking patients about violence.

Arendal LEMC is the 12th largest LEMC in Norway serving a medium-sized Norwegian town and nine surrounding rural municipalities. The generalisability of our results could be questioned with respect to other larger urban areas in Norway, but also with respect to other countries. Many countries have less emphasis on primary care and gate keeping in the organisation of their emergency medical systems than Norway. Three variables ‘No violence suspected, ‘domestic violence suspected’ and ‘Other violence suspected’ were used. The LEMC did not use a validated screening tool for violence, as it was considered too rigid and time consuming in a triage setting. Even though the nurses were instructed to ask all patients about violence, it is likely that there were some interpersonal variations in the threshold for asking about violence as well as follow up questions on violence.

 This could have resulted in some cases of violence remaining undisclosed. Counterintuitively, there is no reason for nurses to avoid questions about violence. Most women do not feel uncomfortable being asked about domestic violence at the EDs [14,15].

This study only registered violence as immediate and acute cause of inquiry to the LEMC. Past exposure of violence often leads to health problems for the victims. Encounters to the LEMC due to sequelae of past exposure to violence, were not included in this study. A question about violence may require follow up questions and demand time, which the nurses may not always have in triage screening work. In the data collection tool, ‘*no violence suspected*’ was pre-filled in as default option.

An important limitation is the proportion of missing cases (25–40% annually). The data indicate significant underreporting of Watchtower data. Reasons include that the nurses in periods with high workload were not able to perform the Watchtower registration, and sometimes it was forgotten. We still believe that adjusting for missing cases gives representative results, and we do not have reason to believe that missing cases were systematically different from the ones captured. Such adjustments were not made when calculating ORs for age and gender. This model for adjusting for underreporting has also been used in previous reports and studies based on Watchtower data [11,13]. We have not identified changes in age and gender distribution among included contacts that indicate significant bias that correlate with underreporting, and we thus consider the adjusted IRRs for disclosed violence as representative.

It was not registered whether it was the patient or another person who made contact. There were no special measures regarding the registration of children. In practice, most often the parents or other caregivers give information on behalf of the child. It would be useful to separately analyse inquiries made patient or by a proxy.

### Findings in relation to other studies

The percentage of suspected victims of violence in this study is lower than usually found in other studies from emergency departments. These studies often find that 1–5% of all treated patient at the ED are victims of violence [16–19]. However, these studies are often based on data from more injury centred clinics, often bypassing primary care OOH-services. The LEMCs handle all types of inquiries directly from the public without any filtering, and a lower proportion of suspected violence is thus expected.

In line with other studies and reports in the field, we found that females were more often victims of domestic violence, while males were more frequently victims of other kinds of violence [16,17,20]. The age of the suspected victims of violence was also in line with other studies in the field [16–18]. However, these studies are on identified victims of violence wherever they are identified at the ED (triage, consultation), while this study registered suspected victims of violence solely at triage level. Thus, many of our findings are in line with other studies from emergency departments and we believe this strengthens the validity of the study.

We found that the proportion of females needing help at the LEMC related to suspected violence was higher than usually found among ED registered victims of violence (50% versus 20–35%) [16,17,20,21]. This may indicate that focus on domestic violence in screening by the triage nurses may have increased the identification of women exposed to domestic violence among all suspected victims. The increased national focus in health institutions on this issue may also have contributed to this increased proportion of females.

There was a high percentage of unspecific RFEs among suspected victims of violence (ICPC-2: A –achapter diagnoses). A previous Norwegian study from all LEMCs providing data to the Watchtower project, found approximately the same proportion of A chapter RFEs (approximately 15%) [22]. Our study also found a high percentage of Z chapter RFEs (social problems) among the suspected victims of violence, while the percentage of this chapter was very low in the previous study (19% versus less than 1%). This is probably related to violence as the most frequent single RFE in our study coded as social problem/traumatic event. It also illustrates that specific RFEs might be difficult to reach at triage level and more specific diagnoses will often be reached during encounters/consultations with medical doctors or/and nurses.

A high percentage of cases were triaged as urgent (yellow) when violence was suspected. This is in line with Norwegian Index [8] where many cases of suspected violence will be triaged as urgent.

An English study found that doctors at an accident and emergency department identified significantly more victims of violence than triage nurses [23]. No proper education on forensic nursing was given prior to the study. Insufficient education in forensic nursing is another potential contributing factor. It may also be challenging to maintain the nurses' attention on screening of domestic violence at first contact difficult to maintain over time [24].

### Meaning of the study

We found that by assessing all first encounters to the LEMC, nurses suspected violence in one in every 311 patients, and domestic violence in one in every 543 female patients. Do these numbers make such screening worthwhile? At first sight, the numbers seem low compared to other studies where 1–5% of patients in OOH primary emergency care report violence. The studies are not directly comparable, and the question remains whether screening in LEMC will reveal violence that would not otherwise have been disclosed.

Ideally, identifying violence at triage level should be done using a short and easy-to-use screening tool and it should be combined with proper education with regular updates. However, it is possible that screening of violence has a higher detection rate during consultation with health care professionals, when higher levels of trust and confidentiality has been established and more time is available than in a triage setting.

Generally, more focus on violence in health care settings is needed, and more training to detect violence in general and the more hidden domestic violence particularly, is necessary [25]. Education in forensic nursing should be strengthened. From 2021, all health care professionals working in emergency health care in Norway are obliged to take a 6–8-hour internet course about abuse and violence, covering several aspects of forensic nursing. We think that early detection and intervention against violence is important. Thus, screening and detection of violence at triage level is an advantage. Future research should investigate the impact of this course on the detection rate of violence, both at triage level and at other levels of emergency care. In a busy LEMC, screening for violence may consume time and potentially displace resources that are needed to handle other patients, some of them with potentially acute life-threatening medical conditions. Screening for violence against partners, as well as elders, and child abuse may be particularly difficult at LEMCs and in emergency departments [26–28].

We believe that screening for violence at triage level is particularly challenging. Time, privacy and patient confidence may be a problem in a triage setting. It is probable that detecting all cases of suspected violence, and especially domestic violence, may be more successful in a calmer and more enclosed environment such as during a consultation with health care professionals where a confidential relationship between the patient and the healthcare provider is established, and more time is available for disclosing sensitive issues.

In this study, we only highlighted the issue violence by including questions about violence at the triage level. Knowledge about following up interventions when the answer is yes, is important. We recommend WHO clinical and policy guidelines (2013) when it comes to domestic violence [29].

## Conclusions

This study finds that when screening at first patient contact with the LEMC, violence is suspected by the triage nurse in 0.3% of all inquiries. Among identified cases, women dominated domestic violence, and men other types of violence.
